# Behavioral Inhibition and Activation Systems, and Emotional Regulation in Individuals With Chronic Musculoskeletal Pain

**DOI:** 10.3389/fpsyt.2018.00394

**Published:** 2018-09-10

**Authors:** Elena R. Serrano-Ibáñez, Carmen Ramírez-Maestre, Alicia E. López-Martínez, Rosa Esteve, Gema T. Ruiz-Párraga, Mark P. Jensen

**Affiliations:** ^1^Facultad de Psicología, Andalucía Tech, Universidad de Málaga, Málaga, Spain; ^2^Instituto de Investigación Biomédica de Málaga, Málaga, Spain; ^3^Department of Rehabilitation Medicine, University of Washington, Seattle, WA, United States

**Keywords:** behavioral inhibition system, behavioral activation system, chronic pain, emotional regulation, cognitive reappraisal, expressive suppression, positive affect, negative affect

## Abstract

Gray's Reinforcement Sensitivity Theory postulates two distinct neurophysiological systems that underlie thoughts, emotions, and behavior: the Behavioral Inhibition System (BIS) and the Behavioral Approach System (BAS). Preliminary research suggests that both systems may play relevant roles in the adjustment of individuals with chronic pain. However, there is a lack of research on the extent to which emotional regulation (i.e., cognitive reappraisal and expressive suppression) mediates the associations between BIS and BAS activation and emotional responses in individuals with chronic pain. The aim of this study was to test a model of the associations between the BIS and BAS, cognitive reappraisal and expressive suppression, and positive and negative affect in individuals with chronic musculoskeletal pain. In total, 516 participants were interviewed. Structural Equation Modeling was used to estimate the associations between variables. The empirical model showed a good fit to the data (χ^2^/df = 1.95; RMSEA = 0.04; GFI = 0.99; AGFI = 0.98; CFI = 0.99). The hypothesized model received partial support. The BIS was associated with cognitive reappraisal and expressive suppression; cognitive reappraisal was associated with negative and positive affect; expressive suppression was positively associated with affect; and the BAS was not associated with the emotional regulation strategies assessed. However, the BIS and BAS were both directly associated with negative and positive affect. The results suggest that individuals with chronic pain with higher BIS activation appear to use greater expressive suppression. Cognitive reappraisal strongly mediated the BIS-negative affect association. The results also suggest that BAS activation may have a weak or inconsistent association with emotional regulation approaches in individuals with chronic pain. These data provide new and relevant information on the potential role of the BIS and BAS as predictors of psychological functioning in individuals with chronic pain. They suggest that the BIS-BAS model of chronic pain may need to be modified to take into account the potential negative effects of BAS activation. The findings suggest that treatments for emotional regulation could potentially reduce the negative impact of chronic pain via BIS.

## Introduction

Chronic pain has been defined as pain that lasts or recurs for more than 3 months beyond the normal recovery time (1). Estimates suggest that 20% of individuals experience pain worldwide (2). In Spain, prevalence is 17% (75% women) (3). Chronic pain is a complex phenomenon that is known to have a negative impact on physical (4, 5) and psychological function (6). Individuals with chronic pain are more likely to report more fear (7), anxiety (8), depressive symptoms (9), and negative mood in general (10) than those without this condition. They are also more likely to report having more posttraumatic stress disorder symptoms (11).

Self-regulation may be especially challenging for individuals with chronic pain because of the association between pain and negative emotion (12). Thus, the negative emotional responses associated with chronic pain may be at least partially associated with difficulties in emotional self-regulation. The ability to regulate emotional experience may contribute to understanding individual differences in the emotion-pain relationship (13). Emotional regulation is a relatively new construct in the chronic pain literature and has been conceptualized as a process by which individuals influence the kind of emotions they have, when they have them, how they experience them, and how they express them (14). Gross (15) has distinguished two emotion regulation strategies: cognitive reappraisal and expressive suppression. Cognitive reappraisal involves anticipating an emotion by evaluating one's thoughts, and then regulating those thoughts in order to experience a preferred emotion. Expressive suppression involves attempts to suppress negative emotions after they have already occurred. Individuals who use reappraisal tend to experience increased positive emotion and decreased negative emotion, whereas individuals who use suppression experience decreased positive emotion and increased negative emotion (16). Thus, the former strategy is viewed as an adaptive emotion regulation strategy and the latter as maladaptive.

A recent systematic literature review (17) described the results of empirical studies on the association between chronic pain and emotional regulation. Most of the studies found indirect associations between emotional regulation and pain that were mediated by psychological factors such as anxiety or negative mood. The results suggested that cognitive reappraisal and expressive suppression do not directly influence the level of pain, whereas expressive suppression negatively impacts anxiety and depression (18), increases catastrophic thinking (19), and worsens daily functioning (20). Based on these results, Koechlin et al. (17) suggested that emotional regulation should be included in theoretical models and in the psychological treatment of chronic pain.

It has been proposed that other factors should be included in psychological explanatory models of chronic pain, such as the Behavioral Inhibition System (BIS) and the Behavioral Approach System (BAS) (21), which have been postulated as neurophysiological systems in Gray's Reinforcement Sensitivity Theory (22, 23). This theory could potentially help to understand the role played by emotions and emotional regulation in adjustment to chronic pain, because these systems are hypothesized to underlie the thoughts, emotions, personality factors, and emotional responses associated with approach and avoidance behaviors (24). It has been hypothesized that the BIS becomes activated in the presence of cues that are associated with the likelihood of punishment and BAS in presence of cues associated with reward. BIS activation then facilitates negative emotions and other responses, such as fear, thoughts of impending doom, and catastrophizing, While BAS activation facilitates impulsivity, hope, joy, and optimism. BIS responses lead to behaviors which decrease the chance of potential punishment (i.e., avoidance behavior) and BAS responses increase the chance of potential reward (i.e., approach behaviors).

Individuals vary in their general trait tendencies in relation to the two systems becoming activated (25), which could explain differences in emotional experience. For example, high BIS activation scores have been shown to predict negative affect (26) and high BAS activation has shown to be related to positive affect (27). Even though there is increasing evidence in support of the association between BIS-BAS and emotional responses, relatively little is known about the processes underlying these effects. It has been hypothesized that BIS and BAS activation could influence emotional responses by affecting emotion regulation strategies.

Consistent with this idea, a positive association has been found between trait BIS activation and emotion regulation difficulties (28). Emotion regulation difficulties have also been shown to mediate the association between BIS sensitivity and anxiety and depression (29). Izadpanah et al. (30) conducted a 5-year longitudinal study in adolescents, finding that BIS activation assessed at a given point in time predicted subsequent maladaptive emotional regulation and anxiety symptoms, and that maladaptive emotional regulation strategies mediated the relationship between BIS and future anxiety. On the other hand, the relationship between the BAS and emotional regulation strategies is less clear. It has been shown that BAS activation predicts subsequent levels of adaptive cognitive emotion regulation strategies (30). However, weak negative associations have also been found between measures of BAS activation and emotional dysregulation (29). Research has suggested that the mediating role of adaptive emotional regulation strategies in the association between BAS activation and emotional responses may be weaker than that of maladaptive emotional regulation strategies (28).

Within the field of chronic pain research, there is a small but growing body of evidence in support of the BIS and BAS having a role in adjustment to this condition. For example, Jensen et al. (31) found an association between higher trait BIS and lower trait BAS scores and a greater frequency of severe headaches. In addition, patients with chronic pain who had higher BIS scores also had more depressive symptoms (32). A positive association has also been shown between BIS activation and pain catastrophizing in a sample of adolescents (33). Elvemo et al. (34) found that individuals with chronic pain tend to have lower hedonic responses to rewards than those without chronic pain. This finding suggests that there is a negative association between pain and BAS activation, which is also consistent with a BIS-BAS model of chronic pain (21). However, we found no study on the association between BIS and BAS and emotional regulation in patients with this condition. To date, no study has assessed the extent to which adaptive and maladaptive emotional regulation mediates the associations between BIS and BAS activation and emotional responses in individuals with chronic pain. Thus, if it could be shown that emotional regulation strategies mediate the impact of trait BIS and/or BAS on emotional responses in individuals with chronic pain, then this information could be included in the development and application of treatments which alter emotion regulation skills, thereby enhancing the benefits of the BAS or buffering the negative impact of the BIS on psychological functioning in individuals with chronic pain.

**Figure 1:**
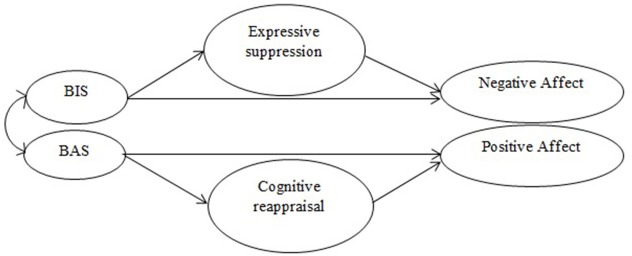
Graphic representation of the study hypotheses.

Given these considerations, the aim of the present study was to better understand the association between BIS and BAS activation and emotional adjustment in individuals with chronic musculoskeletal pain, and the extent to which BIS and BAS activation could influence emotional adjustment by affecting emotional regulation strategies. We address two types of emotion regulation: cognitive reappraisal and expressive suppression (15). We hypothesized that increased BIS activation would be associated with increased negative affect, and that this effect would be at least partially mediated by the positive association between the BIS and emotional suppression. We also hypothesized that BAS activation would be positively associated with positive affect, and that this effect would be at least partially mediated by the association between the BAS and emotional reappraisal. However, previous research on the BIS, BAS, and affect have shown stronger associations between the BIS and emotional regulation strategies than between BAS and these strategies [e.g., (28, 29)]. Thus, we predicted that the mediating role of suppression on the association between trait BIS and negative affect would be stronger than the mediating role of reappraisal on the association between trait BAS and positive affect. Figure 1 illustrates the study hypotheses.

## Methods

### Participants and procedures

The study sample comprised 516 individuals with chronic musculoskeletal pain who met the following inclusion criteria: (1) age, 18 to 65 years; (2) no significant health or psychological problems other than chronic pain (i.e., pain persisting for at least 3 months that is experienced at least 5 or more days per week); and (3) an average pain intensity score of 3 or more on a scale ranging from 0 to 10. A total of 351 participants and 165 participants were recruited from the pain unit of a general hospital and from three fibromyalgia associations, respectively. All participants provided signed informed consent prior to data collection. This study was conducted according to the recommendations of the Comité de Ética del Hospital Regional Universitario de Málaga (Spain), which also approved the study protocol. All participants gave written informed consent in accordance with the Declaration of Helsinki.

### Measures

Each participant completed a battery of questionnaires that were administered in the same order by a psychologist using a semi-structured interview format that lasted about 1.5 h.

### Demographic and pain history information

All participants were asked to provide information on age, sex, marital status, highest educational level completed, and employment status. They also provided information on pain duration and frequency.

### Characteristic pain intensity

Current, highest, lowest, and average pain intensity over the last 2 weeks was assessed using a rating scale that ranged from 0 (“*No pain”)* to 10 (“*Worst pain”*). A mean of these four ratings provided a single composite score of characteristic pain intensity (35).

### Trait BIS and BAS activation

Trait BIS and BAS activation was assessed using the 20-item Sensitivity to Punishment and Sensitivity to Reward Questionnaire [SPSRQ-20; (36)]. The Spanish version of this instrument has two 10-item sub-scales that assess BIS and BAS activity. Participants respond to each item by providing a dichotomous response (“*Yes*” or “*No*”). Example BAS and BIS activation items are “Do you like to put competitive ingredients in all of your activities?” and “Are you often afraid of new or unexpected situations?,” respectively. The internal consistency of the BAS and BIS scales was good (Cronbach's alpha = 0.81) and excellent (Cronbach's alpha = 0.90), respectively.

### Emotional regulation

We used the Spanish version (37) of the 10-item Emotional Regulation Questionnaire [ERQ; (16)] to assess two emotional regulation strategies: (1) cognitive reappraisal (i.e., modifying the emotional impact of the situation by changing thoughts); and (2) expressive suppression (i.e., inhibiting emotional expression once the emotion has occurred). Example cognitive reappraisal and expressive suppression items are “I control my emotions by changing the way I think about the situation I am in” and “When I am feeling negative emotions, I make sure not to express them,” respectively. The cognitive reappraisal scale had excellent internal consistency (Cronbach's alpha = 0.93) and the expressive suppression scale had good internal consistency (Cronbach's alpha = 0.80).

### Positive and negative affect

The 20-item Spanish version (38) of the Positive Affect and Negative Affect Schedule [PANAS; (39)] was used to assess positive and negative affect. Positive affect reflects the extent to which a person feels positive emotion; it is a state of high energy, full concentration, and pleasurable engagement (examples of items are “excited” or “active”). Negative affect reflects the extent to which a person feels negative emotions; it is a general dimension of subjective distress and unpleasurable engagement (examples of items are “distressed” or “scared”). The internal consistency of the positive and negative affect scales was excellent (Cronbach's alpha = 0.92) and good (Cronbach's alpha = 0.88), respectively.

### Statistical analyses

First, we calculated the means, standard deviations, and Pearson's correlations (i.e., descriptive statistics) of the study variables. Next, we tested the assumptions underlying the data (i.e., normality, homoscedasticity, and multicollinearity). Finally, we tested the hypothetical model (Figure 1) using structural equation modeling (SEM). All analyses were conducted using the maximum likelihood estimation method. Model fit was determined by analyzing convergence between several goodness-of-fit indexes (40). The first index used was the Satorra-Bentler chi-square, which adjusts the statistic under distributional violations (41). This statistic is divided by the degrees of freedom to reduce the sensitivity of chi-square to sample size; a value between 0 and 3 indicates that the model has an acceptable fit (42). The second index used was the root mean square error approximation (RMSEA). This index reflects the difference between actual covariance matrices and is fitted with correction for the number of parameters; values adjacent to zero indicate a very good fit and values less than 0.06 indicate a good fit. The next indexes used were the goodness-of-fit index (GFI), which calculates the proportion of variance that is accounted for by the estimated population covariance, and the adjusted goodness of fit index (AGFI), which corrects the GFI based upon degrees of freedom, with more saturated models reducing fit (43). Both indexes can range from 0 (absence of fit) to 1 (perfect fit) and values equal to or more than 0.90 indicate well-fitting models. Finally, we computed a comparative fit index (CFI), which compares the hypothesized model with the null model. The indexes can range from 0 (null fit) to 1 (perfect fit). Values greater than 0.90 indicate a good fit. Based on the modification indexes, changes to the model were made in order to improve fit if they were needed and appropriate (i.e., to only make changes that were theoretically warranted). Statistical analyses were conducted using the SPSS (Windows version 22.0, SPSS Inc., Chicago, IL) and AMOS Graphics (version 22.0; Small Waters Corp., Chicago, IL) software packages.

## Results

### Sample characteristics

The sample comprised 390 women (76%) and 126 men (24%). Mean age was 52 years (SD = 9.27). A total of 168 participants (33%) reported a diagnosis of low back pain, 165 (32%) reported fibromyalgia, 102 (20%) reported limb pain, and 81 (15%) reported a diagnosis of other musculoskeletal pain problems. The participants reported a mean pain duration of 12.82 years (*SD* = 18.08) and a mean pain intensity of 6.40 (*SD* = 1.52) on a scale ranging from 0 to 10. The average time with pain was 6.92 days per week (*SD* = 1.10). Table 1 shows the demographic characteristics of the participants.

**Table 1:** Description of the Study Sample (*N* = 253).

**Variable**	**Percentage(N)**
**MARITAL STATUS**	
Single	9 (47)
Married	62 (321)
Cohabiting	9 (44)
Divorced	15 (81)
Widowed	5 (23)
HIGHEST LEVEL OF EDUCATION COMPLETED^*^	
Fewer than 6 years of education	15 (76)
Primary education	40 (205)
Secondary education	33 (170)
High school	12 (61)
**EMPLOYMENT STATUS**	
Working full- or part-time	37 (192)
Homemaker	16 (81)
Unemployed	21 (106)
Retired	25 (132)
Student	1 (5)

* *Missing values in highest level of education completed (n = 4)*.

### Descriptive analyses and correlations between variables

Table 2 shows the means, standard deviations, and correlations between the study variables. All zero-order associations were statistically significant, except for the associations between BAS and expressive suppression (*r* = −0.02, *ns*) and BAS and positive affect (*r* = 0.03, *ns*). An unexpected weak-to-moderate association was found between BAS and negative affect (*r* = 0.24, *p* < 0.01).

**Table 2:** Means, Standard Deviations, and Correlations Between the Study Variables.

**Variables**	**Mean (SD)**	**1**	**2**	**3**	**4**	**5**
1. BIS (SPSRQ-20)	18.92 (7.65)	–				
2. BAS (SPSRQ-20)	13.50 (4.17)	0.25^**^	–			
3. Cognitive reappraisal (ERQ)	26.36 (8.57)	−0.49^**^	−0.10^*^	–		
4. Expressive suppression (ERQ)	17.84 (5.64)	0.18^**^	−0.02	−0.09^*^	–	
5. Positive affect (PANAS)	30.12 (8.72)	−0.48^**^	0.03	0.54^**^	−0.17^**^	–
6. Negative affect (PANAS)	25.19 (8.18)	0.54^**^	0.24^**^	−0.51^**^	0.19^**^	−0.51^**^

*SPSRQ-20, 20-item Sensitivity to Punishment and Sensitivity to Reward Questionnaire; ERQ, Emotional Regulation Questionnaire; PANAS, Positive Affect and Negative Affect Schedule*.

* *P < 0.05*;

** P < 0.01

### Assumptions testing

In general, all the study variables had normal distributions, with skewness (−0.44 to 0.90) and kurtosis (−0.73 to 2.53) being below the standard cutoff of 3 (44). Correlations between variables did not indicate multicollinearity (43).

### Evaluation of the measurement and structural models

**Figure 2:**
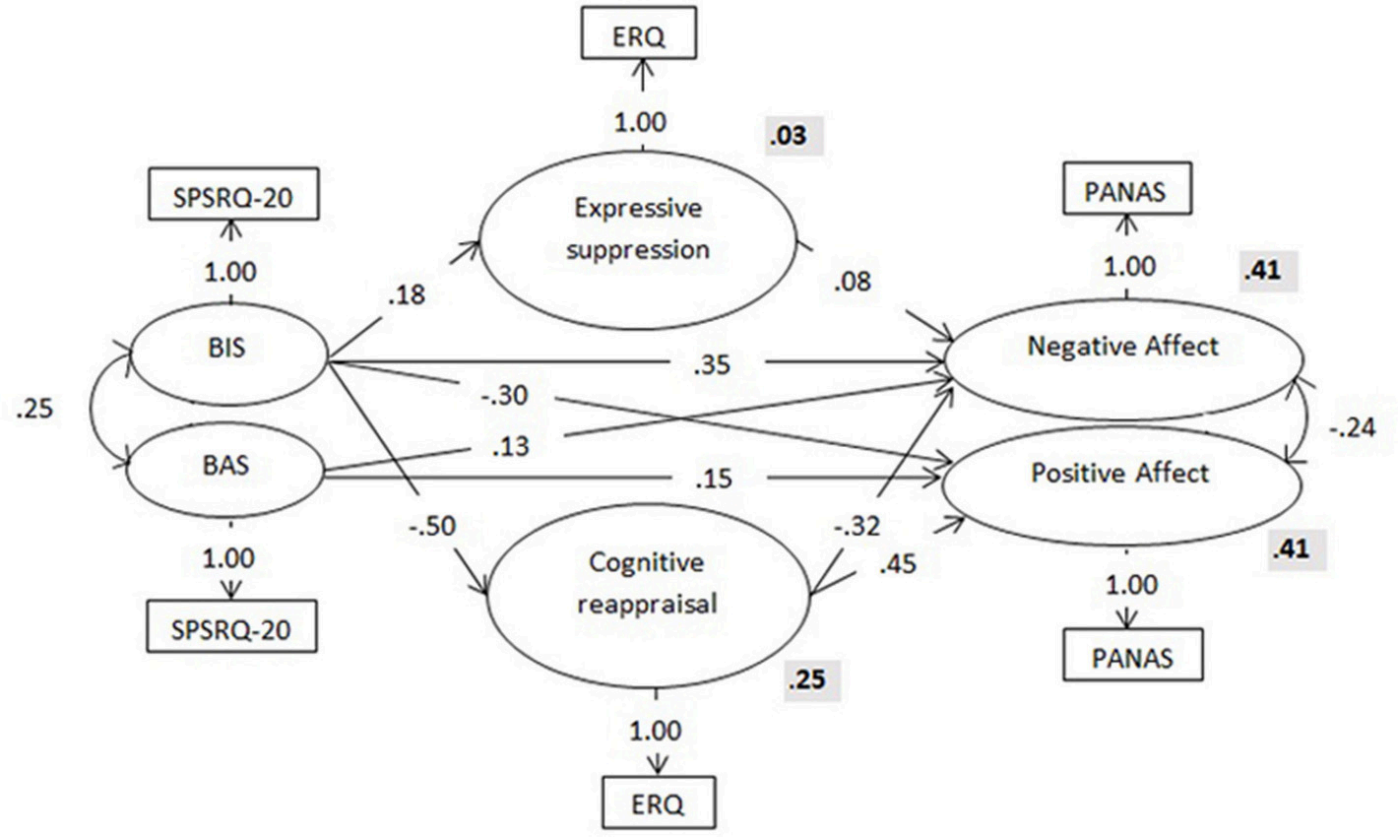
Structure, standardized coefficients (in the arrows) and *R*^2^ values (bold) for the final model. Observed variables are represented by circles and latent variables by square. SPSRQ-20, Sensitivity to Punishment and Sensitivity to Reward Questionnaire; ERQ, Emotional Regulation Questionnaire; PANAS, Positive Affect and Negative Affect Schedule.

The structural equation analysis showed an inadequate fit between the data and the hypothesized model (see Table 3). The modification indexes suggested some possible adjustments that could improve the model fit. We therefore altered the initial model according to these indications. First, we allowed a path between BIS and cognitive reappraisal. We also eliminated the direct path between BAS and cognitive reappraisal because it was non-significant (β = 0.06; *p* = 0.440). We then allowed a path between cognitive reappraisal and negative affect and added a covariance term between the error terms of negative and positive affect. Finally, we allowed BIS to be associated with positive affect and BAS with negative affect. The empirical model showed a good fit to the data [χ2 (*df* = 4, *N* = 516) = 7.81, *p* = 0.099; RMSEA = 0.04; GFI = 0.98; AGFI = 0.98; CFI = 0.99]. Figure 2 shows the final model, the standardized coefficients of each path, and the *R*^2^ values associated with each variable.

**Table 3:** Goodness of Fit Indices From the Structural Equation Modeling Analyses.

**Model**	**χ^2^ (*df*)**	**χ^2^/*df***	**RMSEA (90% CI)**	**GFI**	**AGFI**	**CFI**
Initial model	305.24 (8)	38.15	0.27 (0.242–0.295)	0.88	0.66	0.61
Final model	7.81 (4)	1.95	0.04 (0.000–0.070)	0.99	0.98	0.99

*df, degrees of freedom; RMSEA, root mean square error approximation; GFI, goodness-of-fit index; AGFI, adjusted goodness-of-fit index; CFI, comparative fit index*.

Based on the final model, the BIS yielded four statistically significant path coefficients. There was a direct path from the BIS to both negative affect and positive affect, with higher levels of BIS associated with higher levels of negative affect and lower levels of positive affect. A significant positive path was found from the BIS to expressive suppression (explaining 3% of its variance), which was positively associated with negative affect. A strong negative association was found between the BIS and cognitive reappraisal (explaining 25% of its variance), which was negatively associated with negative affect and positively associated with positive affect. On the other hand, the BAS yielded two statistically significant positive path coefficients; one leading to negative affect and the other to positive affect; that is, higher levels of BAS activation were associated with higher levels of *both* negative and positive affect. Thus, the negative affect (41% of the explained variance) depended on the combined effects of the BAS, the BIS, expressive suppression, and cognitive reappraisal, and the positive affect (41% of the explained variance) depended on the BAS, the BIS, and cognitive reappraisal.

## Discussion

The purpose of the present study was to test a model of the associations between BIS/BAS activation and affect in individuals with chronic musculoskeletal pain, and to investigate the mediating role of emotion regulation strategies in these associations. We hypothesized that BIS activation would be directly and indirectly associated with negative affect, and that the indirect path would be facilitated by the association between the BIS and emotional suppression. Furthermore, we hypothesized that BAS activation would be directly and indirectly associated with positive affect, and that the indirect path would be facilitated by the association between the BAS and emotional reappraisal. However, we also predicted that the direct and indirect associations between the BAS and affect would be weaker than those between the BIS and affect. The model received partial support. To our knowledge, the present study is the first to investigate these relationships in a sample of individuals with chronic pain.

### BIS, affect, and emotional regulation

The results provided support for some of the study hypotheses related to BIS. On the one hand, as predicted, the final adjusted model showed a direct significant association between the BIS and negative affect, which was also mediated by the influence of the BIS on expressive suppression. On the other hand, there was a direct negative association between the BIS and positive affect and an indirect association with positive affect via the mediation path of cognitive reappraisal. This indirect association is inconsistent with the BIS-BAS model of chronic pain (21).

According to the results, individuals with chronic pain with higher BIS activation appear to use greater expressive suppression to regulate their emotions than those with lower BIS activation, which is associated with higher scores on negative affect. As hypothesized, a direct positive association was found between the BIS and negative affect that was stronger than any effects mediated by emotional regulation strategies. These findings are in line with the results of prior studies that found a positive association between BIS activation and emotion regulation difficulties (28) and between emotional suppression and increased negative emotion in undergraduate samples (16). They are also in line with the results of prior studies on individuals without chronic pain (26), in which BIS activation predicted negative affect. Finally, they are also consistent with a BIS-BAS model of chronic pain (21), which hypothesized that BIS activation facilitates the negative emotional responses of anxiety/fear and sadness/hopelessness.

The BIS-BAS model of chronic pain hypothesizes that BIS activation facilitates behavioral inhibition, which has the goal of managing aversive stimuli via avoidance (21). The emotional management strategy of expressive suppression appears to be consistent with the idea of inhibition as an overall coping approach. This strategy is also consistent with the view of pain as a danger signal that simultaneously increases attention to potential threats and suppresses awareness of other motivationally relevant stimuli, thereby restricting the affective space (45). Thus, BIS activation in response to a perceived sense of danger may inhibit the ability to be aware of emotions or manage emotions adaptively. Assuming this proposal is correct, if the BIS generally inhibits behavior as well as emotions, then individuals with chronic pain with increased BIS activation would be expected to be less able to identify and manage their emotions, which would then contribute to more negative affect over time. Consistent with this idea, individuals with chronic pain tend to have increased levels of maladaptive emotional regulation patterns, such as experiential avoidance (46, 47) and alexithymia (28, 48). In fact, a positive association has been found between BIS activation and experiential avoidance in non-chronic pain individuals (49), although the association between BIS and alexithymia remains unexplored. Nevertheless, an association has been found between alexithymia and the anterior cingulate cortex (50), which plays a prominent role in response inhibition (51).

Although the following association was not included in the initial hypothesis, a strong negative association was found between BIS and cognitive reappraisal, which is an emotional regulation strategy thought to be adaptive (16, 17). It is noteworthy that this relationship was stronger than that found between the BIS and expressive suppression. Thus, a novel finding is the strong mediation effect of cognitive reappraisal on the BIS-negative affect relationship. This finding is in contrast with the results of previous research in other populations without pain, which did not find significant associations between the BIS and cognitive reappraisal (30). A positive association has been found between the BIS and pain catastrophizing (33), which is characterized by constant negative rumination about the pain experience. Previous longitudinal studies in adolescents have shown that the association between BIS and emotional problems, such as depression or anxiety symptoms, is mediated by maladaptive cognitive emotional regulation (30, 52). This finding is consistent with the results of the present study. A possible explanation for these unexpected results is that in individuals with chronic pain, the behavioral inhibition facilitated by the BIS may also interfere with the individuals' ability to focus on (“approach” or face) their thoughts, thus making cognitive reappraisal more difficult. This possibility is consistent the studies that have shown that patients with chronic pain use thought suppression as a regulation strategy, which is associated with more anxiety/depression and helplessness/hopelessness (8, 53). It is also in line with experimental research that has shown that suppression applied to pain thoughts, sensations, and emotions was a strategy that promotes increased distress ratings (54). In any case, more empirical research on this topic is needed to determine if our unexpected result is unique to the general population with chronic pain or only to the study sample.

### BAS, affect, and emotional regulation

In contrast to the study hypotheses, no association was found between the BAS and the emotional regulation strategies assessed. Although we predicted that the BAS would play a weak role in overall emotional regulation and affect, we also hypothesized that there would at least be some significant associations. However, these associations were not found. Our results are also inconsistent with those of previous studies, which have found that BAS activation predicted higher levels of adaptive cognitive emotion regulation (30) and that have found a small but statistically significant negative association between BAS activation and emotion regulation difficulties (29). However, the results of the present study suggest that BAS activation may have a weak or inconsistent relationship with emotional regulation approaches in individuals with chronic pain. This suggestion is in line with the results of studies on adults from the general population, which showed that the association between BAS activation and emotional responses was weaker than the association between these responses and BIS activation (28). Further research is needed to help clarify when and if the BAS plays a role in emotional regulation in pain populations.

The results of the present study are mixed regarding the hypothesized direct association between the BAS and affect. We found a direct association between the BAS and *both* negative and positive affect. The positive association of the BAS and positive affect is consistent with previous research (26, 27) as well as with the BIS-BAS model of chronic pain (21), which hypothesizes that BAS activity underlies and facilitates “active” affective responses, such as hope, joy, excitement, and anger. However, we did not predict the positive association between the BAS and negative affect. This is a novel finding that is inconsistent with previous research, although associations have been found between low trait BAS activation and depressive symptoms (55), dysfunctional impulsivity (56), and bipolar disorder (57). Several explanations are possible: (a) this finding may only apply to the general population with chronic pain or only to the study sample; (b) it may be related to the specific measures of the BAS and affect used in this study; or (c) it may be due to a combination of these factors. In relation to this issue, some studies have found different associations between several BAS sub-domains (i.e., Reward Responsiveness, Drive, and Fun Seeking) and functional outcomes (28, 58). The present study used a measure that only assessed the general BAS activation. If we had been able to assess the BAS subdomains, then positive associations may have only been found between a BAS subdomain subset and negative affect.

In any event, the present results suggest an association between positive and negative outcomes and BAS activation in the context of chronic pain. If these results can be replicated, then it would be important to take these potentially negative effects into account and modify the BIS-BAS model of chronic pain (21). Additional research is needed to determine if this finding can be replicated, and to determine the factors that might influence the strength and direction of the associations that have been found.

### Clinical implications

The present results and those from previous studies indicate that the BIS and, to a lesser extent, the BAS have a play role in the emotional functioning of patients with chronic pain. They also suggest that emotional regulation strategies mediate the impact of BIS activation on affect, but do not mediate the effect of BAS activation, at least in relation to the emotional regulation strategies assessed in this study. The findings suggest that treatments which teach patients emotional regulation strategies could potentially reduce the negative impact of BIS activation on adjustment to chronic pain. This approach could lead to less negative affect and greater positive affect in these patients. In particular, the present findings support cognitive reappraisal as a potentially relevant emotional regulation strategy for individuals with chronic pain. This emotional regulation strategy is targeted by cognitive therapy (59) and hypnotic cognitive therapy (60), and is potentially a mechanism by which these two treatments have their beneficial effects.

However, patients may also benefit from treatments that encourage greater emotional expression. Consistent with this idea, there is preliminary evidence in support of the benefits of emotional disclosure for individuals with fibromyalgia (61). The aim of this treatment is to encourage the patient to provide details about emotionally stressful facts or experiences, using reflections and labeling to encourage the participant to experience all the emotions related to that situation.

The present results also suggest that it might be useful to reduce the negative affect that could be associated with relative levels of BAS activation. Behavioral activation is a treatment approach that could address this goal (62). In line with the BIS-BAS model of chronic pain (21), a simple increase in physical activity has been hypothesized to have beneficial effects on positive emotions and adaptive BAS-related beliefs. Behavioral activation can increase engagement in social, recreational, and/or vocational activities. It may therefore also contribute to increased positive emotions through environmental reinforcement (63).

## Limitations and conclusions

The present study has a number of limitations which should be taken in to account when interpreting the findings. First, the study used a cross-sectional design, which makes it impossible to evaluate causality regarding the associations found between the study variables. It would be useful to conduct research on these associations using a longitudinal design. Second, this study only used self-report methods to collect the data. Due to shared method variance, this approach may have artificially increased the strength of some of the associations found. It would be of interest to conduct research on the role played by measures of BIS and BAS as predictors of subsequent observed behavioral and emotional responses as measured more objectively via observation assessments. Third, as mentioned, the measure of BAS activation used in this study only provides a total score of overall BAS activity. For example, as measured on the BIS/BAS scale (58), different associations may have been found between BAS subdomains and emotional regulation and positive affect. Future research should examine this possibility. Fourth, the sample of men was smaller than that of women. Although previous studies shown a higher prevalence of chronic pain in women and the number of men exceeded that required in a SEM analysis, this must be considered when interpreting the results. Finally, only two strategies of emotional regulation were assessed. Future research should examine the potential mediating role of other emotional regulation strategies in the impact of the BIS and BAS on functioning in individuals with chronic pain [e.g., by using the Difficulties in Emotion Regulation Scale [DERS; (64)].

Despite these limitations, some of the strengths of this study include the large sample of individuals with chronic pain and the use of SEM analysis. In addition, the findings provide novel and relevant information on the potential role of BIS and BAS as predictors of psychological function in individuals with chronic pain. We found that the BIS was associated with expressive suppression and cognitive reappraisal, and that cognitive reappraisal was the most relevant strategy for emotional regulation in individuals with this condition. Thus, it may be useful to train individuals with chronic pain in adaptive emotional regulation strategies to buffer the negative impact of the BIS. The present findings suggest that it could be particularly effective to help individuals with high levels of BIS activation to identify and replace irrational or maladaptive thoughts. In line with this idea, the results suggest that the BIS-BAS model of chronic pain may need to be modified to take into account the potential negative effects of BAS activation. More research on this possibility is warranted.

## Availability of supporting data

All data are available to any qualified researcher upon request to the corresponding author.

## Author contributions

AL-M, CR-M, and RE contributed to the design and implementation of the study. GR-P helped to supervise the project. GR-P and ES-I collected the data. ES-I processed the experimental data, performed the analyses, and drafted the manuscript. MJ conceived and supervised the manuscript. All authors discussed the results and contributed to the final version of the manuscript.

### Conflict of interest statement

The authors declare that the research was conducted in the absence of any commercial or financial relationships that could be construed as a potential conflict of interest.

## References

[1] Merskey H Bogduk N Classification of Chronic Pain. 2nd ed. Seattle, WA: International Association for the Study of Pain Press (1994).

[2] Goldberg DS Summer JM. Pain as a global public health priority. BMC Public Health (2011) 11:770–5. 10.1186/1471-2458-11-770 21978149 PMC3201926

[3] Dueñas M Salazar A Ojeda B Fernández-Palacín F Micó JA Torres LM. Nationwide study of chronic pain prevalence in the general Spanish population: identifying clinical subgroups through cluster analysis. Pain Med. (2015) 16:811–22. 10.1111/pme.12640 25530229

[4] Bousema EJ Verbunt JA Seelen HAM Vlayen JWS Knottnerus JA. Disuse and physical deconditioning in the first year after the onset of back pain. Pain (2007) 130:279–86. 10.1016/j.pain.2007.03.024 17467902

[5] Verbunt JA Huijnen IPJ Köke A. Assessment of physical activity in daily life in patients with musculoskeletal pain. Eur J Pain (2009) 13:231–42. 10.1016/j.ejpain.2008.04.006 18547847

[6] Shuchang H Mingwei H Hongxiao J Si W Xing Y Antonius D. Emotional and neurobehavioural status in chronic pain patients. Pain Res Manag. (2011) 16:41–3. 10.1155/2011/825636 21369540 PMC3052406

[7] Zale EL Lange KL Fields SA Ditre JW. The relation between pain-related fear and disability: a meta-analysis. J Pain (2013) 14:1019–30. 10.1016/j.jpain.2013.05.005 23850095 PMC3791167

[8] Lerman SF Zvia R Brill S Hadar S Golan S. Longitudinal associations between depression, anxiety, pain, and pain-related disability in chronic pain patients. Psychosom Med. (2015) 77:333–41. 10.1097/PSY.0000000000000158 25849129

[9] Ferro MA Major depressive disorder, suicidal behaviour, bipolar disorder, and generalized anxiety disorder among emerging adults with and without chronic health conditions. Epidemiol Psychiatr Sci. (2016) 25:462–74. 10.1017/S2045796015000700 26347304 PMC7137593

[10] Zautra AJ Smith B Affleck G Tennen H Examinations of chronic pain and affect relationships: application of a dynamic model of affect. J Consult Clin Psychol. (2001) 69:786–95. 10.1037/0022-006X.69.5.786 11680555

[11] Noel M Wilson AC Holley AL Durkin L Patton M Palermo TM Posttraumatic stress disorder symptoms in youth with vs without chronic pain. Pain (2016) 10:2277–84. 10.1097/j.pain.0000000000000642 PMC5028262 27276275

[12] Hamilton NA Karoly P Kitzman H Self-regulation and chronic pain: the role of emotion. Cognit Ther Res. (2004) 28:559–76. 10.1023/B:COTR.0000045565.88145.76

[13] Hamilton NA Zautra AJ Reich J. Individual differences in emotional processing and reactivity to pain among older women with rheumatoid arthritis. Clin J Pain (2007) 23:165–72. 10.1097/AJP.0b013e31802b4f58 17237666

[14] Gross JJ Emotion Regulation, Handbook of Emotions. 3rd ed. New York, NY: The Guilford Press (2008).

[15] Gross JJ Emotion regulation in adulthood: timing is everything. Curr Dir Psychol Sci. (2001) 10:214–19. 10.1111/1467-8721.00152

[16] Gross JJ John OP. Individual differences in two emotion regulation processes: implications for affect, relationships, and well-being. J Pers Soc Psychol. (2003) 85:348–62. 10.1037/0022-3514.85.2.348 12916575

[17] Koechlin H Coakleyb R Schechterb N Wernerc C Kossowsky J. The role of emotion regulation in chronic pain: a systematic literature review. J Psychosom Res. (2018) 107:38–45. 10.1016/j.jpsychores.2018.02.002 29502762

[18] Hülsebusch J Hasenbring MI Rusu AC. Understanding pain and depression in back pain: the role of catastrophizing, help-/hopelessness, and thought suppression as potential mediators. Int J Behav Med. (2015) 23:251–9. 10.1007/s12529-015-9522-y 26590138

[19] Wong WS Fielding R Suppression of emotion expression mediates the effects of negative affect on pain catastrophizing: a cross-sectional analysis. Clin J Pain (2013) 10:865–72. 10.1097/AJP.0b013e31827da3b5 23370083

[20] Geenen R van Ooijen-van der Linden L Lumley MA Bijlsma JWJ van Middendorp H. The match-mismatch model of emotion processing styles and emotion regulation strategies in fibromyalgia. J Psychosom Res. (2012) 72:45–50. 10.1016/j.jpsychores.2011.09.004 22200522

[21] Jensen MP Ehde DM Day MA. The behavioral activation and inhibition systems: implications for understanding and treating chronic pain. J Pain (2016) 17:529.e1–18. 10.1016/j.jpain.2016.02.001 27052644

[22] Gray JA The Psychology of Fear and Stress. London: Cambridge University Press (1987).

[23] Gray JA McNauhton N The Neuropsychology of Anxiety: an Enquiry Into the Functions of the Septo-Hippocampal System. Oxford: Oxford University Press (2000).

[24] Pickering AD Corr P J. A. Gray's Reinforcement Sensitivity Theory of Personality. The Sage Handbook of Personality Theory and Assessment. London: Sage (2008).

[25] Higgins ET. Value from hedonic experience and engagement. Psychol Rev. (2006) 113:439–60. 10.1037/0033-295X.113.3.439 16802877

[26] Hundt NE Brown LH Kimbrel NA Walsh MA Nelson-Gray R Kwapil TR Reinforcement sensitivity theory predicts positive and negative affect in daily life. Pers Individ Dif. (2013) 54:350–4. 10.1016/j.paid.2012.09.021

[27] Meyer TD Hofmann BU. Assessing the dysregulation of the behavioral activation system: the hypomanic personality scale and the BIS–BAS scales. J Pers Assess. (2005) 85:318–24. 10.1207/s15327752jpa8503_08 16318571

[28] Tull MT Gratz KL Latzman RD Kimbrel NA Lejuez CW Reinforcement sensitivity theory and emotion regulation difficulties: a multimodal investigation. Pers Individ Differ. (2010) 49:989–94. 10.1016/j.paid.2010.08.010

[29] Markarian SA Pickett SM Deveson DF Kanona BB. A model of BIS/BAS sensitivity, emotion regulation difficulties, and depression, anxiety, and stress symptoms in relation to sleep quality. Psychiatry Res. (2013) 210:281–86. 10.1016/j.psychres.2013.06.004 23845417

[30] Izadpanah S Schumacher M Bahr A Stopsack M Grabe JG Barnow S A 5-year longitudinal study of the adolescent reinforcement sensitivity as a risk factor for anxiety symptoms in adulthood: investigating the indirect effect of cognitive emotion regulation. Pers Individ Differ. (2016) 95:68–73. 10.1016/j.paid.2016.02.021

[31] Jensen MP Tan G Chua SM Pain intensity, headache frequency, and the behavioural activation and inhibition systems. Clin J Pain (2015) 31:1068–1074. 10.1097/AJP.0000000000000215 25621428

[32] Jensen MP Sole E Castarlenas E Racine M Roy R Miró J. Behavioral inhibition, maladaptative pain cognitions, and function in patients with chronic pain. Scand J Pain (2017) 17:41–48. 10.1016/j.sjpain.2017.07.002 28850372

[33] Muris P Meesters C van den Hout A Wessels S Franken I Rassin E. Personality and temperament correlates of pain catastrophizing in young adolescents. Child Psychiatry Hum Dev. (2007) 38:171–81. 10.1007/s10578-007-0054-9 17406972 PMC2778719

[34] Elvemo NA Landrø NI Borchgrevink PC Håberg AK. Reward responsiveness in patients with chronic pain. Eur J Pain (2015) 19:1537–43. 10.1002/ejp.687 25766961 PMC6680139

[35] Jensen MP Turner P Romano JM Fisher LD. Comparative reliability and validity of chronic pain intensity measures. Pain (1999) 83:157–162. 10.1016/S0304-3959(99)00101-3 10534586

[36] Aluja A Blanch A. Neuropsychological behavioral inhibition system (BIS) and Behavioral Approach System (BAS) assessment: a shortened sensitivity to punishment and sensitivity to reward questionnaire version (SPSRQ−20). J Pers Assess. (2011) 93:628–36. 10.1080/00223891.2011.608760 21999386

[37] Cabello R Salguero JM Fernandez-Berrocal P Gross JJ A Spanish adaptation of the emotion regulation questionnaire. Eur J Psychol Assess. (2013) 29:234–40. 10.1027/1015-5759/a000150

[38] Sandín B Chorot P Lostao L Joiner TE Santed MA Valiente RM Escalas PANAS de afecto positivo y negativo: validación factorial y convergencia transcultural. Psicothema (1999) 11:37–51.

[39] Watson D Clark LA Tellegen A. Development and validation of brief measures of positive and negative affect: the PANAS scales. J Pers Soc Psychol. (1988) 54:1063–70. 10.1037/0022-3514.54.6.1063 3397865

[40] Byrne B Structural Equation Modeling with AMOS. London: LEA (2010).

[41] Bentler PM. (1990). Comparative fit indexes in structural models. Psychol Bull. (1990) 107:238–46. 10.1037/0033-2909.107.2.238 2320703

[42] Kline RB Principles and Practice of Structural Equation Modeling. New York, NY: Guilford Press (2005).

[43] Tabachnick BG Fidell LS Using Multivariate Statistics. 5th ed. Boston, MA: Allyn and Bacon (2007).

[44] Cam F Saatcioglu O Alexithymia and anxiety in female chronic pain patients. Ann Gen Psychiatr. (2006) 5:13 10.1186/2F1744-859X-5-13 PMC1562423 16911802

[45] Reich JW Zautra AJ Davis M Dimensions of affect relationships: models and their integrative implications. Rev Gen Psychol. (2003) 7:66–83. 10.1037/1089-2680.7.1.66

[46] Costa J Pinto-Gouveia JP. The mediation effect of experiential avoidance between coping and psychocondition in chronic pain. Clin Psychol Psychother. (2011) 18:34–47. 10.1002/cpp.699 21110403

[47] Esteve R Ramírez-Maestre C López-Martínez AE. Experiential avoidance and anxiety sensitivity as dispositional variables and their relationship to the adjustment to chronic pain. Eur J Pain (2012) 16:718–26. 10.1002/j.1532-2149.2011.00035.x 22337134

[48] Saariahoa AS Saariahob TH Mattilac AK Joukamaad M Karukivie M The role of alexithymia: an 8-year follow-up study of chronic pain patients. Compr Psychiatr. (2016) 69:145–54. 10.1016/j.comppsych.2016.05.015 27423355

[49] Pickett SM Bardeen JR Orcutt HK Experiential avoidance as a moderator of the relationship between behavioral inhibition system sensitivity and post-traumatic stress symptoms. J Anxiety Disord. (2011) 25:1038–45. 10.1016/j.janxdis.2011.06.013 21802256

[50] Berthoz S Artiges E Van De Moortele PF Poline JB Rouquette S Consoli SM. Effect of impaired recognition and expression of emotions on frontocingulate cortices: an fMRI study of men with alexithymia. Am J Psychiatr. (2002) 159:961–67. 10.1176/appi.ajp.159.6.961 12042184

[51] Albert J López-Martín S Tapia M Montoya D Carretié L. The role of the anterior cingulate cortex in emotional response inhibition. Human Brain Mapp. (2012) 33:2147–60. 10.1002/hbm.21347 21901794 PMC6870140

[52] Li Y Yun X Zi C. Effects of the behavioral inhibition system (BIS), behavioral activation system (BAS), and emotion regulation on depression: a one-year follow-up study in Chinese adolescents. Psychiatr Res. (2015) 230:287–93. 10.1016/j.psychres.2015.09.007 26386601

[53] Rusu AC Hasenbring M. Multidimensional pain inventory derived classifications of chronic pain: evidence for maladaptive pain-related coping within the dysfunctional group. Pain (2008) 134:80–90. 10.1016/j.pain.2007.03.031 17513052

[54] Masedo AI Esteve R. Effects of suppression, acceptance and spontaneous coping on pain tolerance, pain intensity and distress. Behav Res Ther. (2007) 45:199–209. 10.1016/j.brat.2006.02.006 16569396

[55] Pinto-Meza A Caseras X Soler J Puigdemont D Perez V Torrubia R Behavioural inhibition and behavioural activation systems in current and re-covered major depression participants. Pers Individ Differ. (2006) 40:215–26. 10.1016/2Fj.jad.2006.07.005

[56] Leone L Russo PM Components of the behavioral activation system and function impulsivity: a test of discriminant hypotheses. J Res Pers. (2009) 23:1101–04. 10.1016/j.jrp.2009.08.004

[57] Fletcher K Parker G Manicavasagar V. Behavioral activation system (BAS) differences in bipolar I and II disorder. J Affect Disord. (2013) 151:121–8. 10.1016/j.jad.2013.05.061 23810478

[58] Carver CS White TL Behavioral inhibition, behavioral activation, and affective responses to impending reward and punishment: the BIS/BAS scales. J Pers Soc Psychol. (1994) 67:319–33. 10.1037/0022-3514.67.2.319

[59] Thorn BE Cognitive Therapy for Chronic Pain: A Step-by-Step Guide. New York, NY: Guilford Press (2004).

[60] Jensen MP Ehde DA Gertz KJ Stoelb BL Dillworth TM Hirsh AT. Effects of self-hypnosis training and cognitive restructuring on daily pain intensity and catastrophizing in individuals with multiple sclerosis and chronic pain. Int J Clin Exp Hypn. (2011) 59:45–63. 10.1080/00207144.2011.522892 21104484

[61] Lumley MA Sklar ER Carty JN. Emotional disclosure interventions for chronic pain: from the laboratory to the clinic. Transl Behav Med. (2012) 2:73–81. 10.1007/s13142-011-0085-4 22905067 PMC3419371

[62] Kim EH Crouch TB Olatunji BO. Adaptation of behavioral activation in the treatment of chronic pain. Psychotherapy (2017) 54:237–44. 10.1037/pst0000112 28661164

[63] Harris S Farrand P Dickens C. Behavioural activation interventions for depressed individuals with a chronic physical illness: a systematic review protocol. Syst Rev. (2013) 2:105. 10.1186/2F2046-4053-2-105 24237689 PMC3843584

[64] Gratz K Roemer L Multidimensional assessment of emotion regulation and dysregulation: development, factor structure and initial validation of the difficulties in emotion regulation scale. J Psychopathol Behav Assess. (2004) 26:41–54. 10.1023/B:JOBA.0000007455.08539.94

